# Females Lead Population Collapse of the Endangered Hawaii Creeper

**DOI:** 10.1371/journal.pone.0067914

**Published:** 2013-07-04

**Authors:** Leonard A. Freed, Rebecca L. Cann

**Affiliations:** 1 Department of Biology, University of Hawaii at Manoa, Honolulu, Hawaii, United States of America; 2 Department of Cell and Molecular Biology, University of Hawaii at Manoa, Honolulu, Hawaii, United States of America; Monash University, Australia

## Abstract

Population collapses result from drastic environmental changes, but the sexes may differ in vulnerability. Collapse of the endangered Hawaii creeper (*Oreomystis mana*) at Hakalau Forest National Wildlife Refuge resulted from food limitation associated with increased numbers of an introduced bird (Japanese white-eye, *Zosterops japonicus*), which competes with the creeper for food. Both creeper sexes had stunted bill growth and the greatest change in molt of native species in the community. With a surge in numbers of white-eyes, a recent cohort of adult females had very low survival after breeding, while adult males from the same cohort, and older females and males, continued to have high survival. Lower female survival resulted in a significantly more male-biased adult sex ratio. Recent low female survival was based on a great cost of reproduction, indicated by molt-breeding overlap that was previously avoided, and lower fat during the lengthy fledgling period. The difference in female survival between cohorts was associated with stunted bills from being reared in and then breeding in an increasingly poor food environment. Trend analysis of survey data indicate that the bird is declining throughout the refuge, with males being 72–80% of adults left six years after the white-eye increased. Competition over time was consistent with that previously documented over space on the Island of Hawaii. Adaptive management to recover the bird in this protected area needs to focus on improving both adult female survival and the adult sex ratio.

## Introduction

A population collapse can be distinguished from a long-term decline by the suddenness and rapidity at which it occurs. Many examples exist of population collapses in diverse organisms, with expectation of more to come because of increasingly diverse environmental changes [1]–[5]. Collapses can be documented without regard to sex [6]–[11], in which case it is implicitly assumed that both sexes are declining at similar rates. This assumption may not be true. In some collapses where sex has been considered, adult male mortality was slightly but significantly higher than that of females [12]–[14]. In other studies, there was much higher adult female mortality [15]–[16]. Too few collapses have been documented with sufficient ecological detail to determine the conditions that lead to differential vulnerability of the sexes.

Almost all population declines and collapses of birds have been documented without consideration of sex [7]–[8], [17]–[20]. One study found no difference in adult mortality at the early stages [21]. However, nesting has high energetic expenditures [22], suggesting that females might incur more risks under increasingly challenging conditions. It is important to investigate differential vulnerability of the sexes to environmental change, because the resulting adult sex ratio changes may become more severe without management. Recovery from a population collapse depends on greater survival of the minority sex, but that by itself may simply stabilize and not correct the sex ratio.

Here we focus on declines and potential collapses that are associated with food limitation. Adult males are usually larger than females and would be expected to suffer more when food is limiting, as documented by several studies [12]–[13]. Higher adult male mortality is consistent with avian and mammalian male neonates suffering disproportionally relative to females from food limitation [23]–[25]. However, endangered birds have more extreme male-biased adult sex ratios than do unlisted species [26]. If based on higher mortality of adult females, this imbalance can further the decline that led to endangerment because fewer young will be produced. In general, in species of birds with biparental care, adult females have lower survival than males even in the absence of environmental change, and this is interpreted as a cost of reproduction [27]–[28]. Food limitation associated with an environmental change could therefore affect adult females more than adult males because of this underlying bias in survival.

We explore this possibility with the endangered Hawaii creeper (*Oreomystis mana*), a Hawaiian honeycreeper (Drepanidinae). The bird is endemic to the Island of Hawaii and existed at highest numbers at Hakalau Forest National Wildlife Refuge on the windward slope of Mauna Kea [29]. Food limitation was involved in the observed decline as indicated by lower mass, shorter bills, and shorter legs of young birds [30], and changes in the timing and duration of molt of both hatch-year (HY) birds and adults [31]. There was minor change in HY survival associated with mass, and no change in second-year (SY) survival associated with shorter bills [30], so potential problems with shorter bills in the creeper were carried forward to after-second-year (ASY) adults. The creeper had the greatest change in molt of all eight native species [31]. These changes in the creeper and other species were associated with an increase in numbers of the Japanese white-eye (*Zosterops japonicus*) [30]–[31], an introduced species which competes with many species of Hawaiian birds including the Hawaii creeper [32]. Here we combine demographic, sex ratio, and survey data to document the population collapse of the creeper and to show that it was led by adult females.

## Methods

### Ethics Statement

Mist-netting and bird handling were performed under a protocol approved by the University of Hawaii Institutional Animal Care and Use Committee (00-005-12). The research was approved by the relevant endangered species permits (FREELA-5 through 9, UHMNZA 10–11, TE799001-12 through 15), federal bird banding master permit 21864, state collecting permits (WL-89 through 06), and refuge special use permits (HAK-1-88, SUP-9-93, 56050, and 12516–99014, 030189, 99013, 00009, 01013).

### Study Species and Study Sites

The life history of the Hawaii creeper is typical of small tropical passerines [33]–[35]. Males defend territories around the nest-site [36], and nesting occurs from February through June. The birds have a single two-egg clutch, only females incubate eggs and brood nestlings, and both parents feed nestlings and fledglings [37]. The fledgling period extends for at least three months through most of August [38], and growth of the bill terminates during August [30].

The birds have both high adult survival and long-term pair-bonds [39]. Males provide food to their mates through regurgitation to bring them into reproductive condition, as well as during egg-laying, incubation, and brooding [36]. Females thus depend on their mate for food subsidies for several months of the breeding cycle. They also indirectly depend on their mate for the three to four months of feeding nestlings and fledglings.

**Figure 1:**
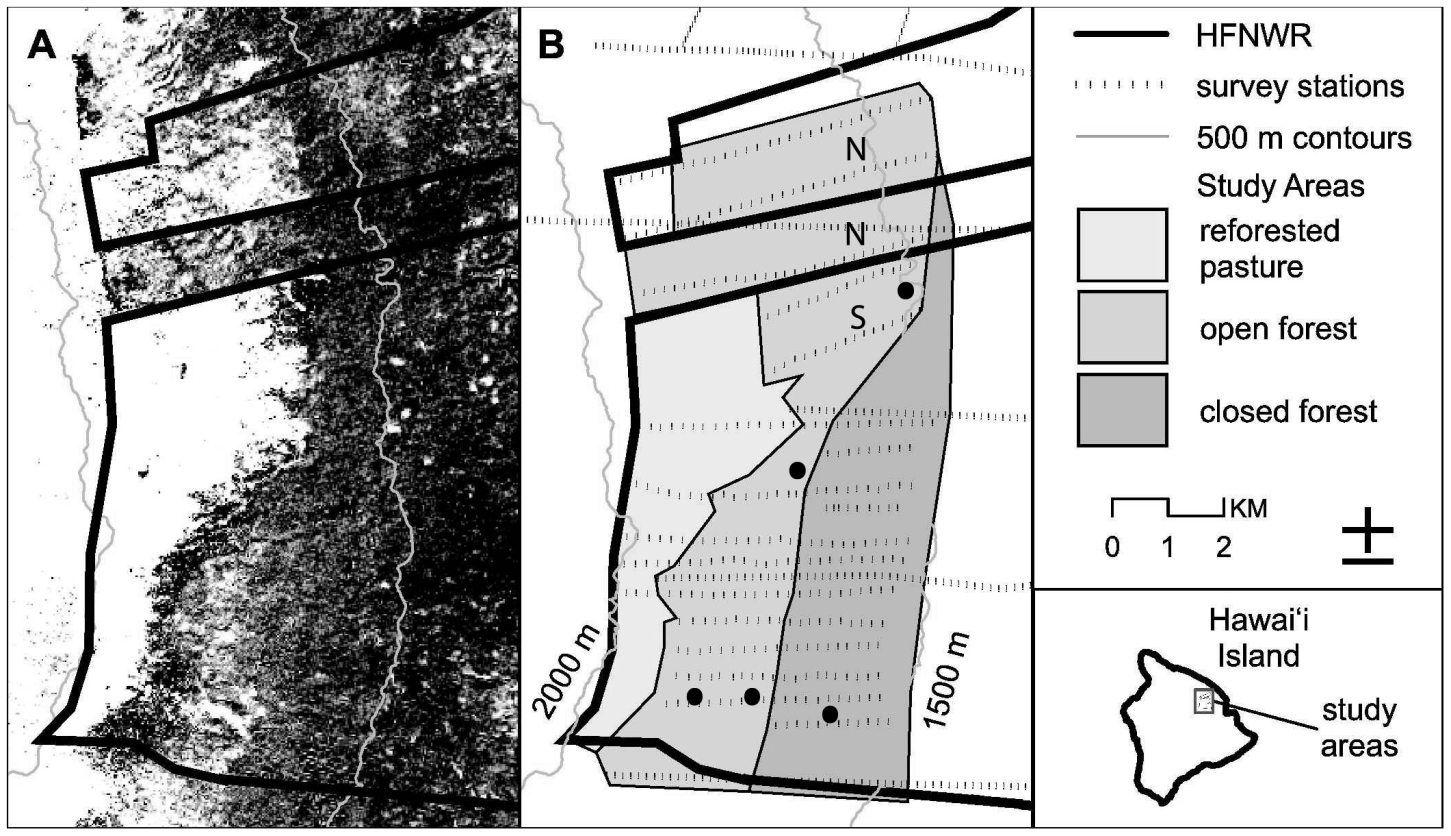
Shown is a map of Hakalau Forest National Wildlife Refuge indicating the restoration, open forest, and closed forest areas (A,B). North is up. Black circles in B indicate study sites in which Hawaii creeper were captured. The southernmost three sites were used for sex ratio. The long-term study site is the westernmost of these and was used for demographic analyses. The increase in Japanese white-eyes was based on propagule pressure from the restoration area above the open forest [48]. Modified from [79], with permission of University of California Press.

The refuge consists primarily of ohia-lehua (*Metrosideros polymorpha*) and koa (*Acacia koa*) trees in a 3373 ha open forest area and a 1998 ha closed forest area (Fig. 1). We captured birds in aerial mist-nets in three different study sites where the creeper existed at high density in the southern end of the refuge [40] (Fig. 1). The long-term site at 1900 m elevation was operated from 1987-mid 2006. Two additional sites in the high density area, at 1770 and 1650 m elevation, were operated during 2004-mid 2006. Survival of the two sexes was estimated from 1987–2006 at the 1900 m site. Adult sex ratio (ASR, proportion females) was estimated annually from year 1998, before the white-eye increase, to 2006 at this site. ASR was estimated as a point value at the 1770 and 1650 m sites during 2004–2006.

Two other sites were operated at distant locations on the refuge where the creeper existed at lower density [40]. The creeper captured at these sites (n = 32), one during 1994–1997 and the other during 2004–2005, were included in the sample used to associate wing length with sex. S-plus 8.2 was used for statistical analysis unless otherwise indicated.

### Processing, Aging, and Sexing Birds

Captured individuals were fitted with a numbered aluminum band and three unique color bands for recognition through binoculars. We measured bill and tarsus length to the nearest 0.1 mm with calipers, tail length to the nearest 0.1 mm with a ruler, and wing length (longest primary straightened and pressed against a wing ruler) to the nearest 0.5 mm. Multiple personnel were trained extensively to take the same measurements [30]. A high resolution photograph was taken of most birds, particularly during 2002–2006. The darkness of the facial mask compared in these digital images generally differs between the sexes [37], and was used to corroborate sex determined by other characters.

HY birds during the breeding season and later in the calendar year could be aged by swollen jaw flanges and whitish feathers where the darker face mask would later appear. As will be documented below, they also have shorter wings than ASY adults, which persist during the first eight months of their second year. SY birds could be aged by molt limits in the wing coverts of their secondary flight feathers [41], as well as by shorter primary wing feathers. ASY birds could be aged by longer wings and more distinct face masks. SY birds between September and December, after they had molted their primary flight feathers, may have been aged as ASY. This is not a problem because SY and ASY birds were included in both the survival and sex ratio analyses.

Most of 171 SY and ASY adults (n = 117) were positively sexed by brood patches in females, cloacal protuberance in males, or by the chromodomain helicase-DNA-binding (CHD) gene [42]. For the latter we used positive passerine controls. We checked for accuracy of sex assigned by CHD gene by comparing wing length with birds sexed by reproduction.

We tested if wing length differed between the sexes, varied between 1987–1999 and 2000–2006, or among study sites with analysis of variance. As will be shown below, wing length alone was a reliable character for sexing most of the 54 individuals captured only during non-breeding months. Only seven of these 54 individuals had wing lengths in the narrow zone of overlap between the sexes. For these individuals, we also used characters of tail, bill, and tarsal length, and darkness of mask to evaluate sex, which was assigned based on closeness to male or female means for the majority of characters. We determined the accuracy of assigning sex to these individuals in the analysis of sex ratio change by repeating the analysis, described below, with and without these individuals.

### Demography and Condition

Mark/recapture analysis was performed using Program Mark [43] on birds recaptured in mist-nets and resighted through binoculars. Mist-netting performed during 2002-mid 2006 (average 4383 aerial mist-net hours per year through 2005) provided ample opportunity to document survival of individuals initially captured before the increase in white-eyes and during 2003 and 2004. An analogous set of control years during 1990–1995 showed that birds captured during 1993 and 1994 survived as well as birds captured earlier in the interval [31]. This means that lower survival of birds captured during 2003 and 2004 was not simply due to fewer years used to document their survival. In addition, 86 days during 2005 were devoted to resighting color-banded individuals by expert observers who identified endangered birds during breeding and later in post-breeding flocks.

The first survival analysis used 72 birds of known sex, including sex assigned without ambiguity by wing length, initially captured during 1987–2002, to evaluate differences in apparent survival and probability of recapture between the sexes. Of particular interest were models that indicated annual variation in survival or recapture through an interaction of these parameters with time or sex. Program Release was used to test for goodness of fit of the best models and indicated adequate fit. The second survival analysis was a cohort analysis in which the 72 birds from 1987–2002 were designated the early cohort. The 38 birds of known sex initially captured during 2003–2006, including individuals with unambiguous wing length, were the late cohort. The cohort models consisted of female change only, male change only, both sexes changing the same, and both sexes changing differently. Because these models are mutually exclusive, we selected the model with lowest AICc.

In addition, we analyzed female survival to at least the following year from the two cohorts captured during the same years 2003 and 2004. For late cohort females, survival was estimated from their initial capture. For early cohort females, survival was estimated from their first recapture or resighting during 2003 or 2004. We compared survival of the two groups of females during the same years with a test of proportions.

We documented breeding success by capture of fledglings and juveniles in mist-nets. Success is essential for knowing if female mortality was associated with breeding and therefore linked to a cost of reproduction. We used mist-net hours from March to December and tested for differences in capture rates between 1999 to 2002 and 2003 to 2006 with a t-test.

We investigated condition of females and males during the last month of nesting in June through October, the month after termination of parental care. We compared furcular fat levels of males and females during the early and late cohort years. Scores were no fat, trace of fat, or partial fill. We compared the proportions of males and females that had partial fill by cohort years using a logistic regression with sex, cohort, and the interaction. The interaction could indicate sexual conflict [44] or otherwise indicate greater vulnerability of females. We also investigated molt-breeding overlap of females captured during June and July as a potential cost of reproduction. A test of proportions determined if more females were captured molting during June or July with an active brood patch during the 2003–2005 period than during 1987–2002. In addition, we compared the prevalence of chewing lice (Phthiraptera) in both sexes to determine if higher female mortality was associated with ectoparasites.

### Change in Adult Sex Ratio

We estimated the number of adult (SY and older) males and females that were present on the 1900 m study site each year from 1998 through 2006. For a long-lived bird such as the Hawaii creeper, individual birds were included in the ASR during multiple years, creating problems for statistical analysis of changes in ASR. However, sex ratio depends on sex, not the identity of males and females. The issue is whether the ASR during 2005–2006 was more male-biased than during the previous seven years. Given the law of large numbers, satisfied by an average of 22 adults per year, a binomial variable not close to 0 or 1 can be approximated by a normal variable [45]. We thus coded a contrast by subtracting the 2005–2006 ASR from each year 1998 through 2004, and evaluated the contrast through a one sample t-test of the differences, using between-year variation in ASR to evaluate the contrast of differences. We tested the accuracy of the sex assignment by comparing the contrasts using birds of known sex, birds of known and assigned sex from non-overlapping wing length, and all birds. We also compared the point estimates of ASR in the 1770 and 1650 m study sites during 2004–2006 with that of the 1900 m site during 2005–2006 using a test of proportions.

### Population Collapse

Estimates of density for 1988–2007 were from annual point counts along transects in the open forest area [46] (Fig. 1). The year 1987 was eliminated because of initial outlier values. We have previously shown, using piecewise regression, that the Hawaii creeper was declining since 2000, but not significantly, in the context of the entire native bird community [47]. The white-eye increase began in 2000 and reached its new stepwise level in 2002 [48]. Here we determine if the creeper declined significantly after the white-eye increase. We averaged the density of 2001 and 2002 as the starting point of the collapse from 2002 to 2007, the last year that density data are available. This controlled for potential outlier values at the start of the series analyzed. We conducted a similar analysis during 1999–2007 for the closed forest area (Fig. 1). We also show the white-eye increase in both areas from [48] and compare the timing and extent of increase of the white-eye and decline of the creeper.

### Changes in Creeper in Relation to White-Eye

Potential asymmetric competition between the creeper and the white-eye was documented by changes in bill lengths. We tested the differences in bill length of male and female creeper and white-eyes during 1987–1999 and 2000–2006 with an analysis of variance that included species, sex, and time period as effects with all interactions. Greater stunting of creeper bills, with other evidence of competition, would indicate asymmetric competition between the species.

We also compared the prevalence of non-normal molt in the creeper during 2004 with the two neighboring years on each side in relation to white-eye capture rates. The year 2004 on the 1900 m study site had a negative residual in a regression of white-eye captures on mist-net hours [21], and was an anomalous year of community-wide low prevalence of non-normal molt [31]. For the creeper we subtracted the 2004 prevalence from that of the other four years and performed a single sample t-test on the arcsine-transformed differences to determine if prevalence of non-normal molt was lower during 2004.

## Results

### Aging and Sexing

HY birds have shorter wings that persist until the second month of their first prebasic molt which started midway through their second year (mean difference of 4.3 mm for sample of 13 birds with wing length measured during HY or early SY, and later in age as ASY birds; paired *t*_12 = _11.4, *P*<0.0001). We could thus distinguish three age classes: HY, early SY, and ASY birds combined with late SY birds.

**Figure 2:**
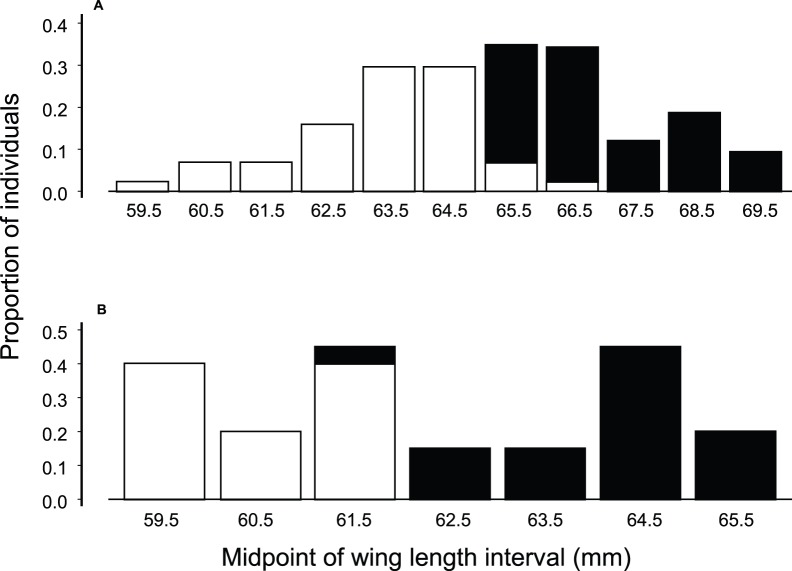
Wing length of male and female Hawaii creeper are portrayed. **A**. Histogram of wing length of female (open bars) and male (filled bars) that were sexed by reproduction or CHD gene. Sample sizes include 44 females and 75 males from all study sites that had completed their first prebasic molt of primary flight feathers late in their second year or their prebasic molt when three years of age or older. **B**. Wing length of second year birds before their first prebasic molt that were sexed similarly. Sample sizes include nine females and 19 males. This figure is associated with Table 1.

We sexed birds accurately based on wing length. Wing length differs significantly between ASY sexes (male mean 66.7, female mean 63.2 mm), and did not vary between time periods or study sites (Table 1). A cut-off of 65 mm distinguished 117 adults positively sexed as males from females with just minor overlap between the sexes (Fig. 2A). We sexed SY birds by wing length because male wings were on average 3.7 mm longer than female wings (Welch modified *t*_24.5_ = 8.64, *P*<0.0001). A cut-off of 61 mm distinguished SY females from SY males with minor overlap (Fig. 2B). All 29 birds sexed by CHD had appropriate wing lengths, suggesting accuracy in measuring wing length and no error in molecular sexing.

**Table 1:** Analysis of variance of wing length in known-sex adults in 5 different study sites and during two time periods (1987–1999 and 2000–2006).

Factor	DF	Sum of squares	Meansquares	F statistic	P-value
Sex	1	346.06	346.06	186.27	<0.0001
Site	4	12.60	3.15	1.70	0.16
Time period	1	4.18	4.18	2.25	0.14
Sex by site	4	6.87	1.72	0.92	0.45
Sex by period	1	0.03	0.03	0.10	0.90
Residuals	107	198.79	1.86		

Of 38 individuals of unknown sex in the three study sites at the southern end of the refuge, seven were in the zone of overlap of wing length. All were resolved based on a combination of facial mask, bill, tarsus, and tail. Three were females, and four were males. We may have overestimated females because they only accounted for 6.8% of birds with wing length 65 and 2.3% with length 66, so the expectation would be no more than one of the seven being females by chance alone.

### Demography and Condition

**Figure 3:**
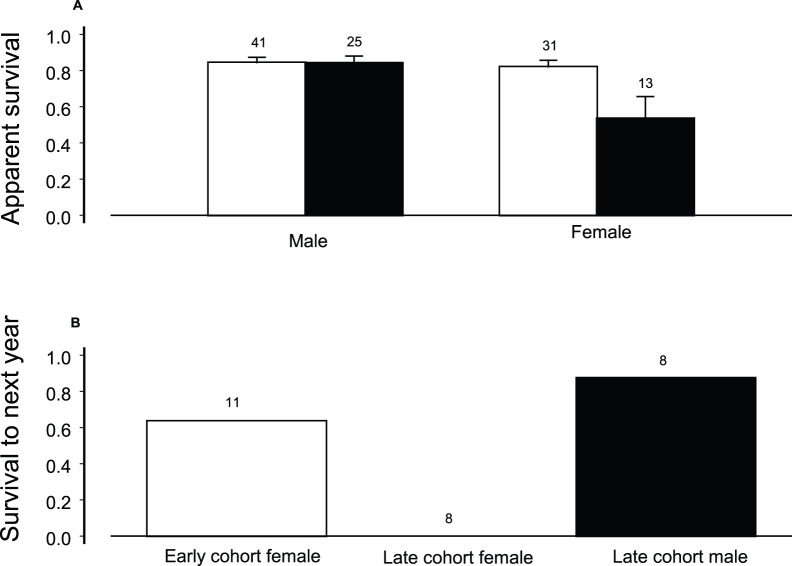
Survival of male and female Hawaii creeper are compared. **A**. Apparent survival with one standard error of Hawaii creeper males and females initially captured during 1987–2002 (white bars) and initially captured during 2003–2006 (black bars). This portion of the figure is associated with Tables 2 and 3. **B**. Survival to the following year of early cohort females recaptured during 2003 or 2004 (white bar), and late cohort males initially captured during those years (black bar). No late cohort ASY females initially captured during those years survived.

Mark/recapture analysis of 72 adults captured in the 1900 m site during 1987–2002 indicated that both sexes had high apparent survival, with slightly but not significantly higher male survival (Fig. 3A, Table 2). There was little or no support for models with survival that varied with time, involved a sex and time interaction, had constant probability of recapture, or had recapture vary with sex or sex and time interaction (Table 2). This suggests, for the sample size, the absence of environmental variability strong enough to influence adult survival over the 15-yr time period. Of the 48 birds that survived, 34 of 40 (72%) were both recaptured and resighted with binoculars. These 72 birds were the early cohort, while the 38 birds initially captured at that site during 2003 to 2006 were the late cohort.

**Table 2:** Mark-recapture analysis of 72 birds initially captured during 1987–2002^1^.

Model	AICc	ΔAICc	Wt	Par.	Deviance	Male *Φ* (se)	Female *Φ* (se)
*Φ*(.) *p*(t)	479.85	0.0	0.745	20	310.74	0.84 (0.03)	0.84 (0.03)
*Φ*(s) *p*(t)	482.00	2.14	0.255	21	310.31	0.85 (0.03)	0.82 (0.04)
*Φ*(.) *p*(.)	496.97	17.12	0.00+	2	369.14	0.83 (0.03)	0.83 (0.03)
*Φ*(.) *p*(s)	497.96	18.10	0.00+	3	368.06	0.83 (0.03)	0.83 (0.03)
*Φ*(t) *p*(.)	498.41	18.56	0.00+	20	329.30		
*Φ*(s) *p*(.)	498.55	18.70	0.00+	3	368.66	0.84 (0.03)	0.81 (0.04)
*Φ*(s) *p*(s)	499.91	20.06	0.00+	4	367.92	0.84 (0.03)	0.82 (0.04)
*Φ*(t) *p*(s)	500.77	20.92	0.00+	21	329.09		
*Φ*(t) *p*(t)	506.27	26.42	0.00	37	288.43		
*Φ*(.) *p*(s*t)	518.97	39.11	0.00	39	294.60	0.83 (0.03)	0.83 (0.03)
*Φ*(s) *p*(s*t)	521.96	42.11	0.00	40	294.26	0.85 (0.03)	0.82 (0.04)
*Φ*(s*t) *p*(.)	536.49	56.64	0.00	39	312.12		
*Φ*(s*t) *p*(s)	539.65	59.80	0.00	40	311.95		

1 Abbreviations: s, sexes are distinguished; t, time dependent apparent survival (*Φ*) or probability of recapture (*p*); dot (.) no sex or no time dependence; s*t, interaction between sex and time.+represents models with less than 0.001 weight. se = standard error.

The female change only model received strongest support in cohort analysis (Table 3). Females in the late cohort had lower survival than males while males had very slightly lower survival (Fig. 3A, Table 3), although there was some support for a model with lower female survival but slightly higher male survival (Table 3). The only late cohort female to survive a year was a SY female captured in 2004 that survived to 2005. Of the 11 birds of both sexes that survived, two of five were both recaptured and resighted with binoculars, not significantly different from the early cohort (test of proportions, *P* = 0.08). Considering survival as a binomial variable, females from the early cohort, alive during 2003 or 2004, survived better than the late cohort females (test of proportions, *P* = 0.02), as did late cohort males (test of proportions, *P* = 0.003) (Fig. 3B).

**Table 3:** Cohort analyses of 72 birds captured during 1987–2002 (early cohort), and 38 birds captured during 2003–2006 (late cohort)^1^.

Model	AICc	ΔAICc	Wt	Par.	Dev.	Male *Φ* (se)	Female *Φ* (se)
♀change only	522.68	0	0.66	22	320.358	0.85 (0.03)	0.83 (0.04) to 0.56 (0.12)
♀♂ change different	525.17	2.48	0.19	23	320.355	0.846 (0.03) to 0.85 (0.08)	0.83 (0.04) to 0.56 (0.12)
♂change only	526.97	4.28	0.08	22	324.643	0.85 (0.03) to 0.89 (0.08)	0.81 (0.04)
♀♂change the same	527.12	4.44	0.07	22	324.795	0.85 (0.03) to 0.76 (0.08)	0.83 (0.04) to 0.76 (0.08)

1 *Φ* represents apparent survival, accompanied by standard error (se).

**Figure 4:**
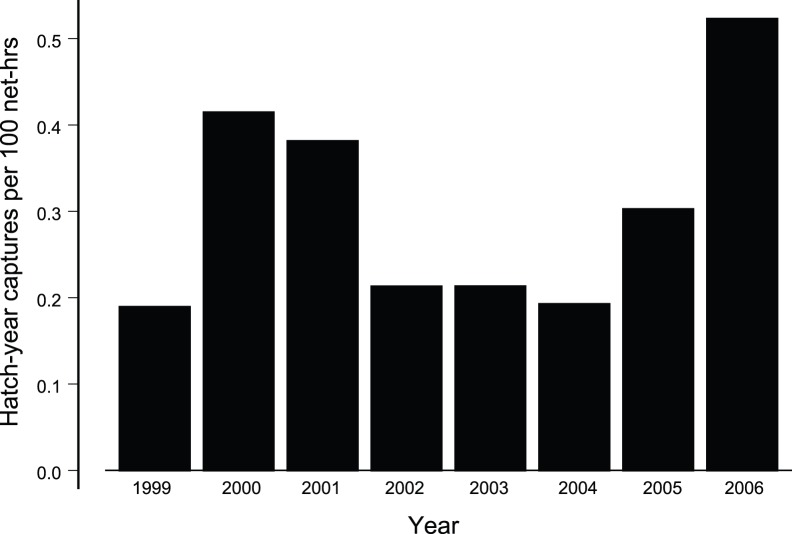
Breeding success of the creeper during 1999–2006 indicated by capture rate of fledglings per net hour is shown.

Breeding success did not differ between cohorts (Fig. 4). Standardized capture rates of HY birds during March -December between 1999–2002 and 2003–2006 did not differ (Welch-modified *t*_4.13_ = 0.93, *P* = 0.42). The similar HY capture rate implies that late cohort females nested successfully.

**Figure 5:**
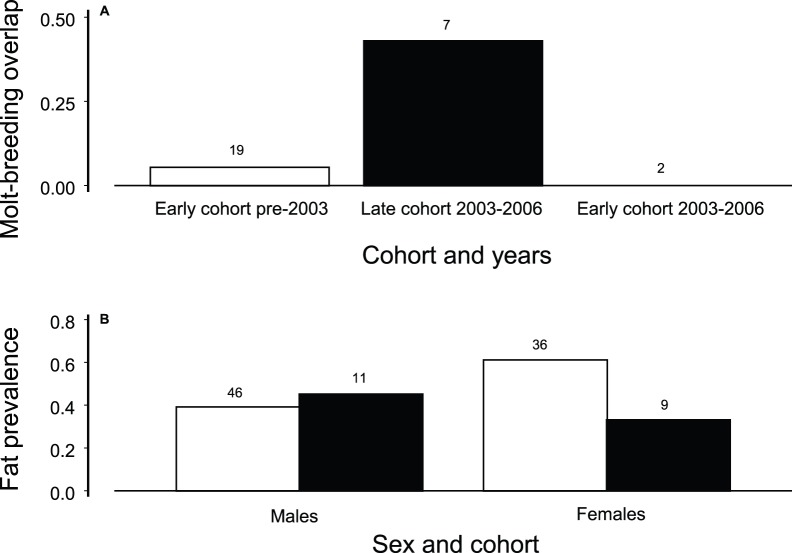
Changes in condition of birds are revealed. **A**. Proportion of females with molt-breeding overlap during June or July during early cohort (white bar) and late cohort (black bar) years. **B**. Differential changes in fat of males and females from the early cohort (white bars) and late cohort (black bars). This portion of the figure is associated with Table 4.

Female creeper had changes in body condition (Fig. 5). Molt-breeding overlap was restricted to late cohort females captured during June and July and was previously very rare in early cohort females captured during the same months before 2003 (Fig. 5A, test of proportions, *P* = 0.03). Fat levels had a marginally significant interaction where the proportion of males with partial fill slightly increased while that of females decreased (Fig. 5B, Table 4). Finally, 0.47 of 32 males had chewing lice compared to 0.24 of 25 females (test of proportions, *P* = 0.13).

**Table 4:** Analysis of deviance of fat levels between male and female creeper during the fledgling period and one month later between time periods 1987–1999 and 2000–2006.

Factor	Df	Deviance	Residual Df	Residual Deviance	P-value
Null			92	128.91	
Sex	1	0.29	91	128.63	0.59
Period	1	0.05	90	128.58	0.82
Sex by period	1	3.82	89	124.76	0.05

**Figure 6:**
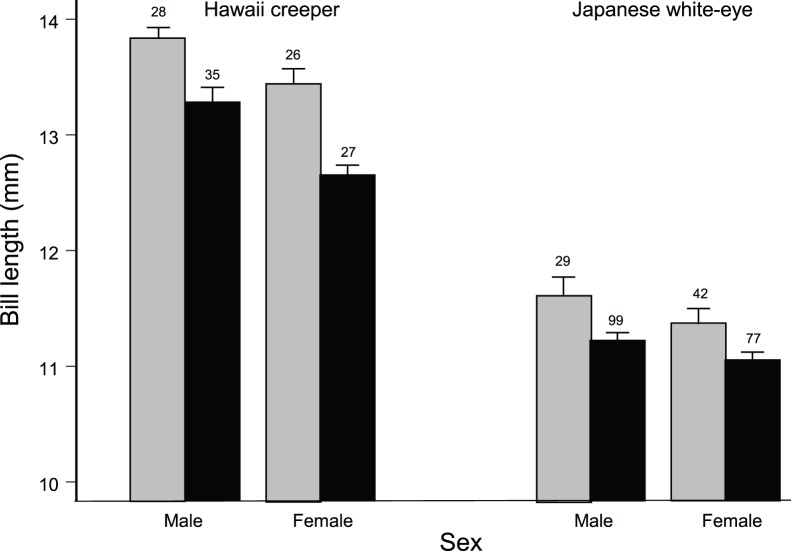
Changes in mean bill length of male and female creeper and white-eyes, with standard errors. Light grey bars represent birds captured during 1987–1999, black bars during 2000–2006. This figure is associated with Table 5. Note the sample sizes which indicate the increase in white-eyes on the 1900 m site.

Hawaii creeper bills were longer than white-eye bills and male bills were longer than female bills for each species (Table 5). Both species had stunted bill length, but the species by time period interaction indicates that greater stunting occurred in the creeper (Fig. 6, Table 5). Bills of Hawaii creeper females were intermediate between those of Hawaii creeper males and white-eye males (Fig. 6). With stunted growth, the size ratio between female creepers and male white-eyes decreased from 1.16 to 1.13. The difference in numbers of the competitors between the two time periods at the 1900 m site was highly significant (*Χ*_3_^2^ = 20.1, *P* = 0.0002). In particular, the number of male white-eyes with bill lengths closer to female creeper more than tripled during the increase (Fig. 6). This greater number was achieved with almost identical mist-net hours operated during 2000–2006 as 1987–1999 (19,004 vs. 19,163).

**Table 5:** Analysis of variance of bill length of creeper and white-eye with respect to sex and time period.

Factor	Df	Sum of squares	Mean squares	F statistic	P-value
Species	1	304.27	304.27	626.79	<0.0001
Sex	1	4.53	4.53	9.34	0.002
Period	1	23.30	23.30	48.00	<0.0001
Species by sex	1	0.72	0.72	1.49	0.223
Species by period	1	4.65	4.65	9.57	0.002
Sex by period	1	0.02	0.02	0.04	0.847
Species by sex by period	1	0.09	0.09	0.18	0.67
Residuals	355	172.33	0.49		

White-eye capture rates were much lower during 2004 than in the neighboring years (21). In 2004, creeper had lower prevalence of non-normal molt than during the combined years of 2002–2003 and 2005–2006 (0.20 *vs* 0.5 and 0.63; *t*_3_ = 4.33, *P* = 0.02).

### Changes in Adult Sex Ratio

**Figure 7:**
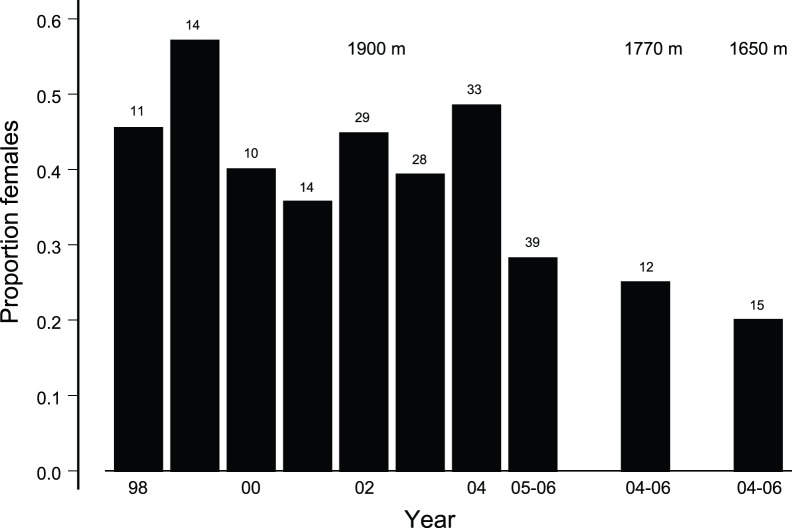
Changes in adult sex ratio during 1998–2006 in the long-term study site at 1900 m, followed by point estimates at 1770 m and 1650 m site used just during 2004–2006. Sample sizes above bars.

The extreme late-cohort adult female mortality among creepers established a more male-biased ASR by 2005–2006 (Fig. 7). Using all adults, that year-set had a sex ratio 0.16 lower than the mean sex ratio during 1998–2004 (*t*_6_ = 6.47, *P* = 0.0006; 0.44 *vs* 0.28 females). Consideration of just birds of known sex or eliminating those that had overlapping wing length did not change the outcome (*t*_6_ = 7.68, *P* = 0.0003; *t*_6_ = 6.57, *P* = 0.0006 respectively). The delay in the change, relative to female mortality, was caused by a higher ratio of unbanded to previously banded females (0.78, n = 14) than males (0.5, n = 20) during 2003 and 2004 that approached significance (*t*_1_ = 9.2 on arcsine transformed proportions, *P* = 0.069). Resighting during 2005 revealed a similar estimate of sex ratio of 0.26 based on 19 detections including only one female not captured in mist-nets that year. Low survival of late cohort females, but high survival of early cohort females, with no change in male survival, made the female subpopulation older than the male subpopulation.

The two lower elevation sites at 1,770 and 1,650 m also had strongly male-biased sex ratios. The 2004–2006 ASR at these sites were similar to the 2005–2006 ASR at the 1900 m site (Fig. 7; test of proportions, *P* = 0.82).

### Population Collapse

**Figure 8:**
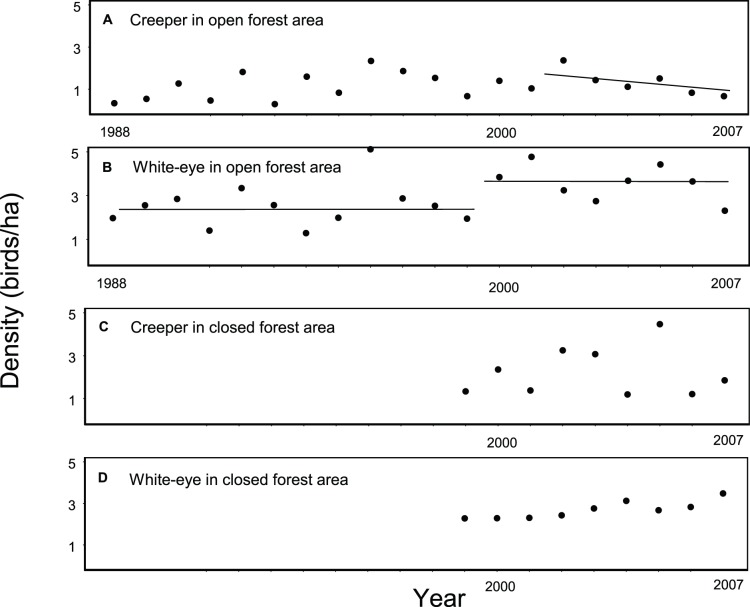
Long term changes in densities of the Hawaii creeper (A and C) and Japanese white-eye (B and D) in the open forest (A and B), and closed forest areas (C and D). Areas are illustrated in Fig. 1. Surveys were not initiated in closed forest area until 1999. The increase in white-eyes in both areas (step-wise in open area and later linear to exponential in the closed area) represents an environmental change [48]. The lower creeper densities in the open area during the first half of the series are not comparable to the collapse between 2001–2002 and 2007. The early density values have higher coefficients of variation than lower densities later in the series, reflecting problems with the data [47]. In addition, the densities cover the entire open forest area, on half of which the creeper exists at very low density [40]. This implies that the collapse may be mainly in the high density area.

The long-term survey data of the Hawaii creeper and Japanese white-eye are shown in Fig. 8. Trend analysis revealed that the creeper population collapsed throughout the 3373 ha open forest area during 2001–2002 to 2007 (Fig. 8A, -0.19 birds/ha/year, *R* ^2^ = 0.75, *P* = 0.03), representing a 63% decline. From 2000 to 2007, the Japanese white-eye had a stepwise increase (48) (Fig. 8B), representing a 44% increase over 1988–1999. There was no other six-year interval for the creeper with a significant decline in the series, although 1996–2001 had a marginally significant decline (*P* = 0.06). However, that was not reflected either in demographic or sex ratio analysis on our 1900 m study site (Table 2, Fig. 7). Decline of the creeper in the 1998 ha closed forest area was not significant (Fig. 8C, –0.27 birds/ha/year, *R* ^2^ = 0.14, *P* = 0.45), but the white-eye was increasing linearly or exponentially in that area (48). During both 2006–2007 white-eye density even exceeded that of the creeper (Fig. 8C,D).

## Discussion

This study provides clear evidence that a significant change in adult sex ratio was caused by differential change in adult demography between the sexes, and documents the first case of a population collapse in a long-lived tropical bird. With the drastic female mortality, the creeper now has a more male-biased sex ratio than most other bird species of concern [26], [49]–[50]. The biased sex ratio is widespread along an elevational gradient in the formerly high density section of the refuge [40], including the closed forest area study site considered more pristine, and is associated with declines that are more widespread in the 3373 ha open forest area. Any population collapse has proximate causes for the mortality (starvation, predation, parasites and disease) and ultimate ecological causes that generate that mortality (climate change, habitat degradation, predators, parasites, pathogens, and competitors). Below we summarize information indicating that the population collapse was associated with starvation, that females suffered an extreme cost of reproduction, and evaluate the ultimate cause of the collapse.

### Proximate Causes of the Collapse

High mortality of female creeper from the late cohort began the population collapse in the long-term 1900 m study site. Females were eventually joined by young birds and males. Many fledglings were produced during the first half of 2006 on the 1900 m site, but 12 of 16 pairs of creeper with fledglings in May lost them by early July, two months before normal termination of parental care [21], [38]. By 2008, the three-syllable fledgling begging call was reduced to one or two syllables. This indicates that the increasing food shortage during 2000–2006, revealed by overall rising prevalence of extended and early molt [31], amplified further during 2006 to 2008. In March and July of 2008, no creeper could be heard or seen at the 1,770 m site during two hour-observation periods, a phenomenon never encountered in 20 years of intense research [21]. Loss of adult males was indicated by the decline in density, because detections of creeper during surveys conducted in March are mainly from singing males.

We infer that creeper losses were due mainly to severe malnutrition. Stunted growth indicates great food shortages during development [51], and stunted growth of creeper involved shorter bills, shorter tarsi, and lower mass [30]. Extended molt can be induced in the laboratory by withholding food [52] as can asymmetric molt of primary flight feathers [53]. Both stunted growth and these changes in molt are unprecedented for the creeper and other native species at this protected locale. Lower female fat during the fledgling period is additional evidence of nutritional stress. Loss of fledglings before the termination of parental care and reduced begging vocalizations are also consistent with severe food limitation. Severe malnutrition and nutritional stress indicate starvation as the proximate cause of mortality.

The molt-breeding overlap with one to two months of additional parental care and the lower fat levels of late cohort females point to a cost of reproduction that led to mortality. This cost in a normally long-lived tropical bird is the most extreme cost of reproduction ever documented [54]–[56]. The molt-breeding overlap was previously avoided by the creeper as it is by most birds [57]–[58]. The cost was likely derived from being reared under poor conditions. Diverse examples exist of fitness consequences later in life of birds and other animals reared under poor conditions [59]–[60]. There is also evidence of differential effects of being reared in a poor environment on the two sexes [61]. Stunted bills of both sexes reflects being reared under poor conditions, and the problem was carried forward to ASY birds because SY survival did not change [30]. Problems in rearing offspring created low fitness in adult females when they eventually attempted to breed.

Sexual conflict over parental care is widely known [44], [62]–[63] and may also have played a role. When one parent does not contribute its share the other parent partially compensates [64]. Males might have adequately subsidized females during incubation and brooding to maintain the breeding attempt, but may not have fully participated during feeding of nestlings and fledglings. Compensating care by females can account for the lower prevalence of partial fat in late-cohort females and the slight increase in prevalence in late-cohort males. The normal bill lengths of early-cohort males and females is associated with normal fat levels and survival. However, sexual conflict cannot account for the eventual decline of males indicated by the survey data.

### Ultimate Causes of the Collapse

Several hypotheses that lead to starvation can be readily dismissed. Three species of introduced rats, a mongoose, and feral cats are nest predators of Hawaiian birds [65], but nest predators could cause starvation only by preventing parents from visiting the nest. However, parents do not feed nestlings at night when the black rat (*Rattus rattus*), the most serious predator for forest birds [65], is active. Avian malaria (*Plasmodium relictum*) might have increased food requirements of affected females as shown for the sex with higher parental investment in other species [66], and in general for cost of reproduction [67]. However, the elevations at which our study was conducted are much too cool for the parasite to develop in the mosquito vector, and malaria is rare at these elevations [68]. There was no sign of climate warming between the years of the early and late cohort birds [31], which could have altered food availability to both white-eyes and creepers. There was also no habitat degradation on the refuge where weedy plants were controlled.

However, introduced insects could have played a role in the mortality of late cohort females as competitors for food. Yellow-jacket wasps (*Paravespula pensylvatica*) consume some of the same arthropod resources as Hawaiian forest birds [69], but they were monitored and controlled on the refuge beginning in the late 1990s and extending into the late cohort years [30]. Introduced parasitoid wasps were present but were more common at lower elevations [70], where the creeper did not significantly decline. The explosive increase in chewing lice that began in 2003 could have increased food requirements of the birds over food that was available [71]. But male creeper had higher prevalence of lice, ruling out ectoparasites. Food limitation, evident as stunted growth and non-normal molt, occurred before 2003 [21], [30]–[31].

Interspecific competition with the introduced Japanese white-eye is the well-supported remaining hypothesis for the collapse. Ever since the Hawaii Forest Bird Survey conducted in the 1970’s [29], the Hawaii creeper has been considered susceptible to competition with the white-eye based on negative correlation in densities between the two species, after adjusting for differences in forest structure between sites [32]. The basis for this susceptibility is that the white-eye overlaps all six foraging substrates used by the creeper, while using 11 additional substrates in the simple ohia-lehua/koa forest [21]. Consistent with the negative correlation in densities, the creeper is extremely rare in the northern portion of the refuge where white-eyes exist at high density [40], [48]. The collapse of the creeper in the southern forested portion of the refuge, which began in 2002, was associated with a white-eye increase, which began in 2000, based on a randomization test [48]. Our study thus documents the negative correlation in densities in the 3373 ha open forest area in real time.

Stunted growth and changes in molt are the mechanism of food limitation from competition. During 2004–2005, creepers had stunted growth in the 1900 and 1770 m sites but normal growth in the 1650 m site, associated with a fivefold higher capture rate of white-eyes in mist-nets in the higher elevation sites [30]. During 2005, prevalence of community-wide non-normal molt was higher in the upper elevation sites, but higher in the 1650 m site in 2006 [31]. This was associated with a decline in white-eyes in the open forest area between 2005 and 2006 but increased density in the closed forest area during those years (Fig. 8B,D). In addition, in our 1900 m site, white-eye captures in mist-nets dropped in 2004 [21], associated with lower prevalence of non-normal molt in the creeper. It is difficult to imagine that another factor could result in this diverse set of changes coincident in space and time. Moreover, the marginal decline of the creeper in the open forest area between 1996 and 2001, but not in our 1900 m site, may have been associated with a short-term spike in white-eye density in 1996 in other portions of the open forest area (Fig. 8B).

The bill lengths of the creeper and white-eye, especially between female creeper and male white-eye, are too similar for stable coexistence. Hawaiian birds evolved with bill length ratios of approximately 1.22 [21], consistent with the theory of limiting similarity which specifies how similar two species can be and still coexist [72], [73]. In this light, the ratio of 1.16, becoming 1.13 with stunting, would lead to increasing competition especially with the explosive increase in white-eyes on the study sites.

Competition theory recognizes that both intraspecific and interspecific competition occur, and that coexistence depends on the relative strength of the two forms. In particular, two species can coexist when they inhibit themselves more than the other species [74]. However, there are many reasons why competition favors the white-eye. First, the overlap between foraging substrates is asymmetric with white-eyes having many additional foraging substrates [21]. Second, white-eyes come from a family of birds renowned for niche diversification [75], even among individuals within a species [76]. These may be the reasons why there was greater stunting of creeper bills than white-eye bills, and why white-eyes did not suffer survival consequences of stunted bill length as did native birds [30]. Third, the increase in white-eyes was maintained by propagule pressure, indicated by white-eye juvenile survival going from 0.28 to 0 during the increase with no change in mass [21]. Lower mass is usually the reason for lower juvenile survival [77]. Thus, increased adult numbers in the open forest study sites and most of the open forest area had to be maintained by propagules from the restoration area, where the white-eye population was growing exponentially [48]. Dominance from adult white-eyes at higher density may have been responsible for dispersal of juveniles to the closed forest area, where the population was growing [48]. Most other native species declined with the creeper in both areas of the refuge, with only the white-eye increasing in both areas [48].

### Conclusions

The future of the endangered Hawaii creeper at Hakalau Forest National Wildlife Refuge is precarious. With adult sex ratios of 72–80% males in the southern portion of the refuge, this is a clear example that survey data taken at face value may be totally misleading. The refuge has likely lost more than 60% of the creeper in the open forest area, with most of the remaining individuals being males, and the increasing density of white-eyes in the closed forest area suggests that it will follow suit. It is ironic that these same data have been used to highlight the Hawaii creeper as a conservation success story, a species increasing on the refuge from management of introduced ungulates and weeds [78]. This assertion is based on single-slope trend analysis [79] for which analysis of residuals indicates significant lack of fit of the model [40], [47]. The current management plan for the refuge emphasizes forest restoration, which we have shown will increase white-eyes and make matters worse for native birds in the forest below [48].

The management which urgently needs to be implemented is to increase adult female survival and restore the adult sex ratio of the creeper. At minimum, this will include controlling Japanese white-eyes. This approach will also provide the opportunity to formally test the competition hypothesis and should help many other declining species [48]. Reduced numbers should increase ASY female creeper survival, and prevent the adult sex ratio from becoming even more male biased. The return to the former more equitable adult sex ratio is much more problematical. An adjustment to the primary sex ratio cannot be expected until females that produce two daughters have higher fitness than those that produce one son and one daughter [80]–[81]. When, as the minority sex, they do have higher fitness through management, this system has the potential to reveal the adjustment expected by sex ratio theory. This is because sex allocation is an adaptation in the related endangered Hawaii akepa (*Loxops coccineus coccineus*), which unfortunately, has been dismantled by competition with white-eyes [41]. However, if no adjustment occurs, captive breeding may be necessary for production of offspring from which females can be released into the wild to restore the adult sex ratio to its former value.
